# Bioinformatics of Metalloproteins and Metalloproteomes

**DOI:** 10.3390/molecules25153366

**Published:** 2020-07-24

**Authors:** Yan Zhang, Junge Zheng

**Affiliations:** 1 Shenzhen Key Laboratory of Marine Bioresources and Ecology, College of Life Sciences and Oceanography, Shenzhen University, Shenzhen 518055, China; junge.zheng@hotmail.com; 2 Shenzhen-Hong Kong Institute of Brain Science-Shenzhen Fundamental Research Institutions, Shenzhen 518055, China; 3 Shenzhen Bay Laboratory, Shenzhen 518055, China

**Keywords:** metal, metalloprotein, metalloproteome, bioinformatics, comparative genomics, evolution

## Abstract

Trace metals are inorganic elements that are required for all organisms in very low quantities. They serve as cofactors and activators of metalloproteins involved in a variety of key cellular processes. While substantial effort has been made in experimental characterization of metalloproteins and their functions, the application of bioinformatics in the research of metalloproteins and metalloproteomes is still limited. In the last few years, computational prediction and comparative genomics of metalloprotein genes have arisen, which provide significant insights into their distribution, function, and evolution in nature. This review aims to offer an overview of recent advances in bioinformatic analysis of metalloproteins, mainly focusing on metalloprotein prediction and the use of different metals across the tree of life. We describe current computational approaches for the identification of metalloprotein genes and metal-binding sites/patterns in proteins, and then introduce a set of related databases. Furthermore, we discuss the latest research progress in comparative genomics of several important metals in both prokaryotes and eukaryotes, which demonstrates divergent and dynamic evolutionary patterns of different metalloprotein families and metalloproteomes. Overall, bioinformatic studies of metalloproteins provide a foundation for systematic understanding of trace metal utilization in all three domains of life.

## 1. Introduction

Biological trace metals refer to those metals which are only needed in small quantities but are essential for normal development, survival, and reproduction of all living organisms [1,2]. These micronutrients include iron (Fe), zinc (Zn), copper (Cu), manganese (Mn), molybdenum (Mo), tungsten (W), nickel (Ni), cobalt (Co), chromium (Cr), vanadium (V), and several other elements, which play vital roles in a wide variety of biological and chemical events. Some metals act as critical cofactors or as important structural components for different enzymes, whereas others can accept or donate electrons in various redox reactions, or regulate biological processes by facilitating the binding of molecules to corresponding receptors [3,4]. The major metalloid, selenium (Se), is also involved in several pivotal cellular processes in both prokaryotes and eukaryotes [5]. Deficiency and excess of these micronutrients may cause abnormalities in development and metabolism or even death [6].

Among all metals, Fe and Zn are indispensable for all or almost all living organisms [7,8]. The utilization of other trace metals is highly diverse and scattered. Cells require metal ions as cofactors for the assembly and activation of metalloproteins. Most metals are directly incorporated into their binding sites on these proteins, whereas a small number of metals must form metal-containing cofactors or complexes before their insertion into target proteins, such as molybdopterin (or named Mo cofactor, Moco) for Mo and cobalamin (vitamin B_12_) for Co [9,10]. Metalloproteins comprise a numerous and diverse group within the proteomes of organisms, which not only catalyze a remarkably wide range of important reactions but also play important structural and regulatory functions [11]. It has been suggested that approximately one third of all proteins require metals for their biological roles and almost half of all enzymes must associate with one or more metal ions [12,13]. The number of metalloprotein families varies greatly depending upon which metal they use. For instance, Zn is estimated to be used by several hundred protein families, whereas less than ten protein families belong to Ni-dependent metalloenzymes [14,15]. In addition, Se exerts its biological functions mainly as the 21st amino acid selenocysteine (Sec, encoded by the codon TGA) in selenoproteins [16]. The presence of metalloproteins necessitates a tight regulation of metal metabolism and homeostasis to maintain the appropriate metal concentration in the cell while avoiding toxicity, which includes transport, delivery, storage, detoxification, and efflux machineries [17,18].

In recent years, due to the rapid development of high-throughput sequencing technologies, the availability of genomic sequences for a huge number of organisms has opened the door for systematic analysis of the occurrence and evolutionary trends of metal utilization in nature. The main achievements of these studies lie in in silico identification and comparative genomics of metalloproteins across the tree of life. A variety of bioinformatic algorithms have been developed for the detection of metalloprotein genes. Moreover, large-scale analysis of the metalloproteome (the complete set of metalloproteins) in different organisms may help us to better understand the utilization and function of trace metals in biology. This review focuses on current computational approaches used for metalloprotein gene prediction as well as relevant tools and resources. We will also introduce recent advances in comparative genomics involving several essential metals to present an integrated and holistic picture of the distribution and evolution of metalloproteins and metalloproteomes in all domains of life.

## 2. Identification of Metalloprotein Genes and Related Resources

### 2.1. Homology-Based Identification of Known Metalloprotein Genes

In the past several decades, a large number of metalloproteins and their functions have been recognized. Therefore, metalloprotein genes may be identified by sequence homology to previously characterized metalloproteins. In general, representative metalloprotein sequences can be used to scan either genomic (using TBLASTN) or protein databases (using BLASTP). Significant hits aligned with the query sequences are potential members of known metalloprotein families. It has been reported that many metalloprotein families either possess both metal-dependent (i.e., strictly dependent on certain metals for their function) and metal-independent (i.e., metal-coordinating residues are partially or completely lost) forms or have evolved to use alternative metals in different organisms [19,20]. Thus, metalloproteins should be further verified by examining the conservation of known metal-binding residues in the corresponding families.

The homology-based method may also be applied to identify selenoprotein genes in genomic datasets. Considering that selenoprotein genes have several highly specific sequence-structural features [16], additional steps are needed for their efficient and correct recognition. Representative selenoprotein sequences derived from previously reported selenoprotein families are used to search against the genomic database using TBLASTN or similar approaches. Homologs containing a putative in-frame TGA codon that aligns the Sec residue in the query are initially considered as selenoprotein gene candidates. As the mechanism underlying Sec biosynthesis and its insertion into selenoproteins has been thoroughly investigated in recent years [21,22], the presence of an essential *cis*-acting structure called Sec insertion sequence (SECIS) element which is located in either 3′-untranslated regions (eukaryotes and archaea) or immediately downstream Sec-TGA codon (bacteria) of selenoprotein mRNAs should then be examined by SECIS prediction tools (see below for details). Furthermore, due to the fact that most known selenoproteins have homologs in which Sec is replaced with a cysteine (Cys) residue [23], all selenoprotein candidates should be searched against the NCBI non-redundant protein database using BLASTP for the presence of conserved Cys-containing homologs. A computational pipeline named Selenoprofiles has been developed to correctly annotate selenoprotein genes belonging to known families in genomic sequences based on homology searches [24].

### 2.2. Methods for Prediction of Metal-Binding Sites and Novel Metalloprotein Genes

In recent years, with the exponential increase in the number of completely sequenced genomes, there is an urgent need to develop bioinformatic algorithms allowing the prediction of new metalloprotein genes or even the search for entire sets of metalloproteins. A number of computational tools and methods have been developed for the prediction of either metal-binding sites in proteins (particularly for Zn and Fe) or metalloprotein genes in different organisms. A list of most of these tools is shown in Table 1.

Several early studies proposed a general protocol taking advantage of known amino acids present in the metal-binding region of metalloproteins and other related sequence-structural features [25,26,27,28]. It combines the concepts of metal-binding domains (sequence profiles) and metal-binding patterns (residues coordinating the metal ion in 3D structures of metalloproteins), and examined their occurrence and correlation in all proteins of organisms. This strategy has been widely used for identification of metalloprotein genes and metalloproteomes for several essential metals (such as Zn, Cu, and Fe) in a variety of organisms from the three domains of life, which provides important clues to protein function and protein-ion interaction [25,26,27,28,29,30,31]. Based on this approach, a software package called RDGB was developed to identify putative homologs of the proteins of interest (such as metalloproteins) in any genome [32].

Zincfinder is a software for the prediction of Zn-binding proteins based on the support vector machine (SVM) method [33]. This predictor identified some unprecedented Zn-binding sites which were further validated through homology modeling. Another SVM- and homology-based algorithm was also developed to improve the performance of Zn-binding site prediction, which could provide higher precision at different recall levels compared to Zincfinder [34].

TEMSP [35] is a structure-based tool to predict Zn-binding sites in proteins. This method may be a significant improvement over existing methods in predicting Zn-binding residues from protein structures with minimum overpredictions. Moreover, TEMSP can reliably predict the Zn-bound local structures and is helpful for functional inference of Zn-binding proteins.

Zincidentifier [36] is an integrative framework which combines sequence, structural, and graph-theoretic network features, followed by a two-step feature selection using a random forest algorithm. This method can not only be an effective tool for accurately predicting Zn-binding sites using structure data, but also provide information on new properties for characterizing Zn-binding sites.

ZincExplorer [37] is a new hybrid algorithm for effective prediction of Zn-binding sites in protein sequences, which is composed of SVM-, cluster-, and template-based predictors. Furthermore, it could infer the interdependent relationships of the predicted Zn-binding sites that bind to the same Zn atom.

ZincBinder [38] is a newly developed SVM-based method for prediction of Zn-binding sites in proteins based on sequence profile information. This tool showed better performance than some existing methods.

ZINCCLUSTER [39] is a novel tool for identification of Zn-binding sites of proteins based on primary sequences or 3D structures. It can predict amino acids interacting with Zn or other metal ions, which is based on the occurrence of significant triplets found in the active sites of metalloproteins. It appears to have higher prediction accuracy than other computational tools such as FINDSITE-metal and SeqCHED.

ZnMachine [40] is a very recently reported method for high-throughput Zn-binding residue prediction by combining several well-designed machine learning models. These models were constructed using different types of sequence profiles and effective features derived from multiple sources and were then combined using a linear equation. This tool demonstrated competitive performance and could become a complementary approach to the existing methods.

HemeBIND [41] is the first specialized SVM-based algorithm used to predict heme (an Fe-porphyrin complex) binding sites by combining several sequence and structural attributes that have distinctly different distributions between heme binding and non-binding residues. A significant improvement in prediction performance has been shown when compared to individual structure-based and sequence-based classifiers alone.

SCMHBP [42] is a scoring card method based tool for predicting and analyzing heme-binding proteins using propensity scores of amino acids and dipeptides. This approach performed well when compared to some typical methods such as SVM, decision tree, and Bayes classifiers, which may improve our understanding of heme-binding proteins.

Previously, an integrative penalized linear model based on machine learning approach has been reported for the prediction of iron-sulfur (Fe-S) proteins, which demonstrated higher sensitivity and a good level of specificity when compared to motif-based approaches [43]. MetalPredator [44], another web-based tool to predict Fe-S proteins from protein sequences, was also developed to process complete proteomes with high-precision prediction, providing new resources for an extensive exploration of Fe-S proteomes on a large-scale.

Besides the above mentioned methods focusing on specific metals, several bioinformatic tools have been developed for general metal-binding site prediction. For example, MetSite [45] identifies metal-binding sites using sequence profiles and approximate structural information from predicted models; FINDSITE-metal [46] is a threading-based algorithm designed to detect metal-binding sites in modeled protein structures, which combines structure/evolutionary information with machine learning results; SeqCHED [47] is developed for predicting metal binding sites from protein sequences based on remote homology detection between template and target protein; MetalDetector [48] can predict metal binding sites for proteins having novel folds and does not rely on 3D structure similarity; MIB [49] is a web server built to predict different types of metal-binding residues using the fragment transformation method, which also provides metal ion-docking visualization functions to generate the predicted metal ion-bound 3D structure after prediction. Very recently, deep neural networks have also been applied for prediction of metal-binding abilities of certain amino acids such as histidine (His) and Cys residues, offering a powerful technique for large-scale functional genomic screening [53].

It should be admitted that in silico identification of metalloprotein genes is a quite challenging issue as current knowledge gained from experimental work makes it almost impossible to identify the complete metalloproteomes in most organisms. Since different bioinformatic methods have different advantages and disadvantages in capturing key aspects of metalloproteins, an alternative approach is to apply a variety of complementary tools for the prediction of metalloprotein genes. It has also been suggested that many metalloproteins in prokaryotes and humans have not been identified yet [54,55], and more efficient and accurate tools are needed to search for additional metalloproteins and metal-binding features.

Compared to metalloprotein prediction for which highly precise method is still lacking, computational identification of selenoprotein genes and the set of all selenoproteins (selenoproteome) in different organisms is easier and more reliable due to several specific features for Sec insertion machinery [21,22]. Several bioinformatic tools have been widely used for selenoprotein gene prediction in different kingdoms [56]. SECISearch3 is currently the most efficient and widely used method for identification of eukaryotic SECIS elements, and is a key component of Seblastian which is a new method for the identification of selenoprotein genes in eukaryotic genomes [50]. SelGenAmic is a gene assembly algorithm for detecting selenoprotein genes from eukaryotic genomes and has been used to identify selenoproteins in metazoans [51]. The method bSECISearch is used for the prediction of selenoprotein genes in bacteria based on a predefined sequence/structural pattern and several other constraints [52]. Additional SECIS-independent algorithms were also developed, which employ Cys-containing proteins in large protein databases to search against nucleotide sequence datasets for selenoprotein genes [57]. Both SECIS-dependent and SECIS-independent approaches have shown excellent performance in identifying all or almost all selenoprotein genes in different genomes.

### 2.3. Metalloprotein Databases

Integration of information about metalloproteins from various resources (such as public nucleotide/protein databases and literatures) provides the basis for further understanding their utilization and extensive roles. In recent years, several metalloprotein databases have been built up, such as MDB, Metal-MACiE, MetalPDB, and some other databases. A list of these databases is shown in Table 2.

MDB (the Metalloprotein Database and Browser) [58] is a web-accessible resource for exploring metalloproteins, which provides quantitative information on protein metal-binding sites from structures available at the Protein Data Bank (PDB). MDB also offers tools for the examination of patterns in metal-binding sites, and for the prediction of metal sites from new protein structures.

Metal-MACiE [59] is a publicly available database that contains the information on the roles of metals in catalytic mechanisms of metalloenzymes. This database can be used to improve our knowledge of the chemistry underlying metal-dependent catalysis.

dbTEU (DataBase of Trace Element Utilization) [60] is a manually curated protein database, which contains ~16,500 protein sequences of all known transporters and metalloproteins for several trace elements (such as Cu, Mo, Co, Ni, and Se) in a variety of organisms from all three domains of life. It also offers interactive tools for searching and browsing organisms, metalloprotein families, and other related information.

Mespeus [61] is a useful database for investigating the variety of metal ion-protein interactions. It lists experimentally established geometry of metal protein interactions with a user-friendly interface, and can also contribute to the modeling process.

MetalPDB [62] is a valuable resource of metal-binding sites detected in the 3D structures of biological macromolecules, which represents such sites in the form of Minimal Functional Sites. This database also provides extensive statistical information on structural aspects associated with individual metals, giving a better understanding of the diversity in biochemical roles of metals.

SelenoDB [63] contains full annotations of selenoprotein genes in approximately 60 animal genomes, including alternative transcripts and a worldwide catalog of genetic variations in human selenoprotein genes. It is an important resource for medical and evolutionary studies on Se.

ZincBind [64] is a newly developed database of all known Zn-binding sites from PDB. It contains more than 16,000 unique Zn-binding sites, which are then organized into groups and families. It also has a friendly web interface for users to browse, search and download data of interest. This resource is useful to researchers working on Zn-binding site/protein prediction and modeling, and will be expanded with new data in future.

## 3. Comparative Genomics of Metalloproteins and Metalloproteomes

**Figure 1:**
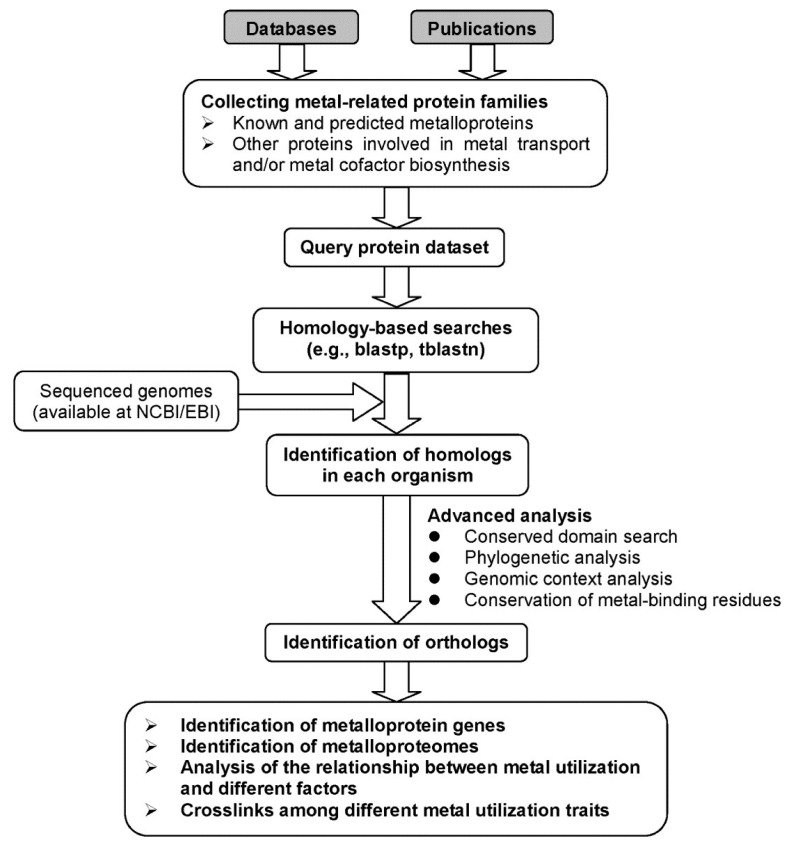
A general diagram for comparative genomic analysis of metal utilization.

Comparative genomics provides a powerful tool for investigating evolutionary changes of genes, pathways and other characteristics along various lineages [65]. Based on comparative genomic approaches, we may better understand the use of metalloproteins and metal-dependent processes in living organisms. However, identification and quantification of complete metalloproteomes for most metals is currently impossible [66,67]. Even so, analysis of the majority of them in genomic databases can still greatly improve our understanding of the utilization and function of metals and their variations across species during evolution. In addition, analysis of genes involved in metal uptake, homeostasis, and metal-containing cofactor biosynthesis may assist in identification of metal utilization trait (i.e., the ability to use certain metal) [67,68,69]. A general procedure for comparative genomics of metal utilization is shown in Figure 1. In the following sections, we mainly focus on metalloproteins and discuss recent progress on comparative genomic analyses of metalloproteins for several important metals.

### 3.1. Zinc and Iron

Zn and Fe are the two most commonly used trace metals in all organisms. A great number of proteins have been characterized or predicted to use one of the two metals (see references [25,26,30] and several web resources described above for a compiled list of Zn- or Fe-binding protein families). However, because of the widespread and complex use of the two metals, comparative analyses of the occurrence and evolutionary trends of their utilization are still very challenging to handle [69].

Zn is known to contribute to numerous biological processes in living systems, which is a key component present in hundreds of structural proteins, enzymes, transcription factors, and ribosomal proteins [14]. Previously, Zn proteomes (including both Zn-dependent proteins and some other proteins involved in Zn transport and homeostasis) have been predicted in a limited number of prokaryotic and eukaryotic organisms based on Zn-binding domains and patterns extracted from various databases [25,28,29,70]. In general, the number of Zn-binding proteins are positively correlated with the proteome size of an organism. Eukaryotes had a higher proportion (8%~10%) of Zn-binding proteins than prokaryotes (5%~6%). The majority of prokaryotic Zn proteins perform enzymatic catalysis (especially hydrolases) while the eukaryotic Zn proteome is mainly involved in both catalysis and transcription regulation of gene expression, suggesting that Zn-binding transcription factors have evolved to regulate more complex and diverse processes in higher organisms [28,29]. Another study analyzed two major groups of Zn proteins (Zn finger-containing proteins and Zn hydrolytic enzymes) in more than 800 organisms, which revealed that there is a correlation in their changes during evolution related to environmental change [71]. In recent years, comparative genomic approaches have been frequently used to investigate the distribution and diversification of certain Zn-dependent protein families, particularly Zn finger-containing transcription factors such as PRDM, Zic, and several other C2H2-Zn finger protein families, which provides a basis for further research on the origin, function, and evolutionary features of these proteins [72,73,74,75]. With an explosion in genomic resources and the rapidly expanding number of bioinformatic tools in the past decade, a more comprehensive analysis of Zn-dependent proteomes in all kingdoms of life is urgently needed.

Fe is the most abundant transition metal in cells and has a fundamental role in many metabolic processes, such as oxygen transport, electron transfer, nucleic acid synthesis, growth, and many important redox reactions [7]. Besides Fe ions, many proteins may use Fe in the form of heme or Fe-S clusters [7,11]. Due to the complexity and diversity of Fe utilization, it is very hard to identify the complete Fe-dependent proteomes; however, several studies have been performed aiming at the understanding of different groups of Fe-dependent proteins in various organisms. An early bioinformatic study investigated the occurrence of putative non-heme Fe-binding proteins in a small number of prokaryotes and eukaryotes, which demonstrated that extant organisms have inherited the majority of Fe proteome from the last universal common ancestor [26]. Compared to Zn proteome, the Fe proteome constituted a higher fraction of the proteome in archaea (7.1% on average) than in bacteria (3.9%) and in eukaryotes (1.1%). Another computational study compared the distribution of Fe-S proteins in more than 400 prokaryotic organisms with different life styles and found a strong relationship between environmental dioxygen levels and the usage of different Fe-S clusters [31]. Very recently, the complete human Fe proteome was systematically analyzed based on different types of Fe-containing cofactors [30]. About 2% of human genes encode Fe proteins (35%, 48%, and 17% for individual Fe ions, heme, and Fe-S clusters, respectively). Interestingly, genes encoding Fe proteins (especially Fe-S proteins) appeared to be more commonly related to pathologies than all other human genes, suggesting specific features of the physiological role of Fe. In addition, comparative genomic analyses were carried out for investigating Fe metabolism and homeostasis mechanisms in different organisms, such as cytosolic Fe-S cluster assembly machinery [76], heme biosynthesis and uptake machinery [77], and several other protein families involved in Fe transport and storage [78], which provide detailed insights into the composition and evolution of Fe metabolic network.

### 3.2. Copper

Cu is an important activator for several key enzymes participating in fundamental biological processes such as respiration, photosynthesis, and oxidative stress responses. A number of Cu-dependent proteins (cuproproteins) have been characterized in both prokaryotes and eukaryotes. The currently known cuproprotein families are shown in Table 3 (proteins involved in Cu transport and homeostasis are not included).

Comparative genomic studies have been previously carried out to analyze intrinsic features of different cuproproteins or cuproproteomes (the whole set of cuproproteins) in various organisms [27,79,80,81,82]. Two early studies combined known Cu-binding domains and Cu-binding patterns to explore the occurrence of Cu proteins (including both cuproproteins and some other proteins involved in Cu transport and homeostasis) in a limited number of sequenced genomes [27,79]. The proportion of Cu-binding proteins was small when compared to that of Zn or non-heme Fe proteins. Eukaryotes have expanded the Cu proteome inherited from the last common ancestor of all organisms by evolving new Cu domains and reusing old domains for novel functions.

**Figure 2:**
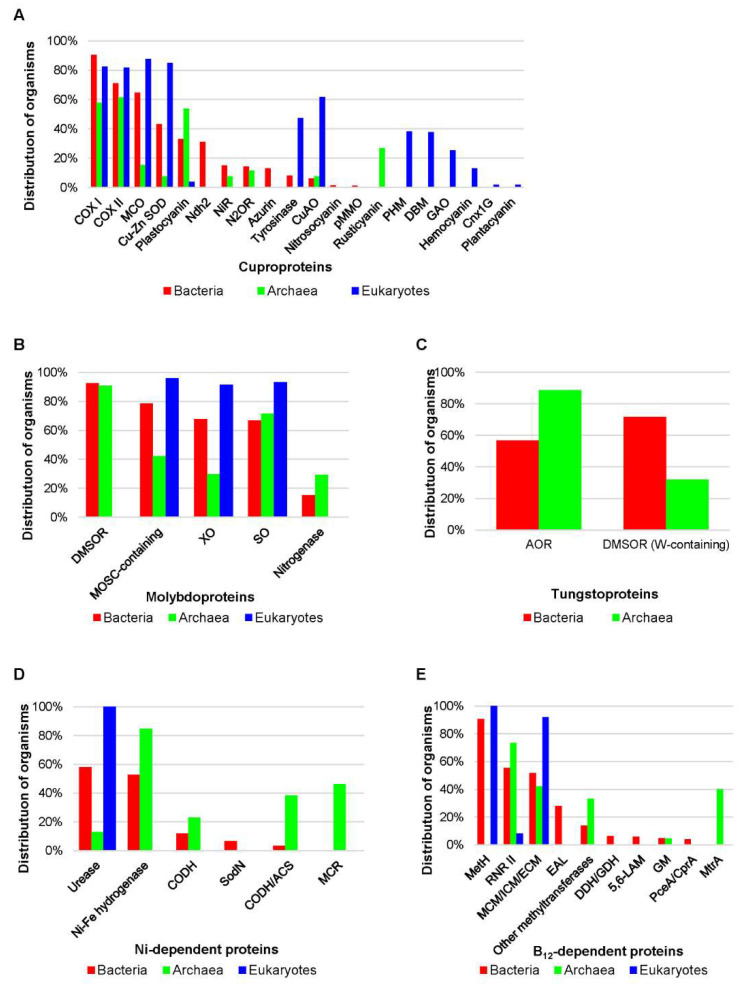
Distribution of metalloprotein families in the three domains of life. (**A**) Distribution of cuproproteins in Cu-utilizing organisms; (**B**) Distribution of molybdoproteins in Mo-utilizing organisms; (**C**) Distribution of tungstoproteins in W-utilizing organisms; (**D**) Distribution of Ni-dependent proteins in Ni-utilizing organisms; (**E**) Distribution of B_12_-dependent proteins in Co-utilizing organisms. COX I, cytochrome c oxidase subunit I; COX II, cytochrome c oxidase subunit II; MCO, multicopper oxidase; Cu-Zn SOD, Cu-Zn superoxide dismutase; Ndh2, NADH dehydrogenase 2; NiR, nitrite reductase; N2OR, nitrous oxide reductase; CuAO, Cu amine oxidase; pMMO, particulate methane monooxygenase; PHM, peptidylglycine α-hydroxylating monooxygenase; DBM, dopamine β-monooxygenase; GAO, galactose oxidase; DMSOR, dimethylsulfoxide reductase; XO, xanthine oxidase; SO, sulfite oxidase; AOR, aldehyde:ferredoxin oxidoreductase; CODH, carbon monoxide dehydrogenase; SodN, Ni-containing superoxide dismutase; CODH/ACS, acetyl-coenzyme A synthase/decarbonylase; MCR, methyl-coenzyme M reductase; MetH, methionine synthase; RNR II, B_12_-dependent ribonucleotide reductase class II; MCM, methylmalonyl-CoA mutase; ICM, isobutyryl-CoA mutase; ECM, ethylmalonyl-CoA mutase; EAL, ethanolamine ammonia lyase; DDH/GDH, diol/glycerol dehydratase; 5,6-LAM, D-lysine 5,6-aminomutase; GM, glutamate mutase; PceA/CprA, B_12_-dependent reductive dehalogenase; MtrA, methyltetrahydromethanopterin:coenzyme M methyltransferase subunit A. Data used to generate this figure are available in the supplementary information of [81,83].

Some other studies provide more detailed information about cuproproteins in the three domains of life [67,80,81]. Cytochrome c oxidase subunits I (COX I) and II (COX II) are the most widely distributed cuproproteins in prokaryotes (Figure 2A). Multicopper oxidase (MCO), Cu-Zn superoxide dismutase (Cu-Zn SOD) and plastocyanin families were also found in many prokaryotes, whereas the occurrence of tyrosinase, nitrosocyanin, Cu amine oxidase, and particulate methane monooxygenase seems to be quite limited. Except for cuproproteins that were exclusively present in individual kingdoms (e.g., azurin in bacteria and rusticyanin in archaea), significant difference in the distribution of most cuproproteins was observed between archaea and bacteria (Figure 2A). On the other hand, only half of prokaryotic cuproprotein families could be found in eukaryotic organisms, and the latter have the capacity to evolve new cuproproteins such as galactose oxidase, hemocyanin, and plantacyanin. MCO, COX I, COX II, and Cu-Zn SOD were the most abundant cuproprotein families in eukaryotes, while the distribution of some cuproproteins appeared to be phylum-specific, e.g., hemocyanin in arthropods and plantacyanin in land plants. A recent comparative analysis of the presence of hemocyanin in different myriapod species suggests that these proteins have divergent evolutionary patterns in different myriapod taxa [82]. Further analysis of prokaryotic cuproproteomes revealed that larger cuproproteomes were mainly present in Alphaproteobacteria, Betaproteobacteria and Euryarchaeota/Halobacteriales. The largest bacterial and archaeal cuproproteomes reported to date were detected in several *Sinorhizobium* species (*S. medicae* and *S. meliloti*, 22 cuproprotein genes) and *Haloarcula marismortui* (25 cuproprotein genes), respectively [81]. In eukaryotes, land plants possessed the largest cuproproteomes, especially *Oryza sativa* containing 78 cuproprotein genes). It is interesting that larger cuproproteomes were mainly found in organisms living in oxygen-rich environments, which is consistent with the idea that proteins evolved to use Cu following the oxygenation of the Earth [80,81,82]. Because previous studies relied only on a limited number of organisms, future research is needed to update the distribution and evolution of cuproproteins/cuproproteomes using a much wider range of sequenced genomes belonging to different clades.

### 3.3. Molybdenum and Tungsten

Mo is required for the activity of a number of molybdoproteins that catalyze diverse reactions in the metabolism of carbon, nitrogen, and sulfur compounds [9]. With the exception of Fe-Mo-containing nitrogenase, Mo needs to be bound to a specific pyranopterin moiety to form Moco, an active compound at the active site of all molybdoproteins [84]. Some prokaryotes (such as hyperthermophilic archaea) use W to replace Mo, which is bound to the same pyranopterin to form tungstoproteins [85]. A list of known molybdoprotein and tungstoprotein families is shown in Table 3. Each family may contain a variety of enzymes [86,87]. It has been suggested that MOSC (Moco sulfurase C-terminal domain)-containing proteins are new members of the sulfite oxidase (SO) family due to similar structures for Mo-binding domains [86]; however, the lack of significant sequence similarity between them may challenge such an alternative classification approach [88].

Several comparative genomic studies have been conducted to explore the distribution and evolution of Mo utilization trait and molybdoproteins in all domains of life, which give preliminary indications of how this transition element is used by different organisms [81,89,90]. Very recently, the occurrence of all known molybdoprotein families in nearly 6000 sequenced prokaryotes and eukaryotes was analyzed, which presents a much more comprehensive view of the evolutionary trajectories of molybdoproteins in nature [83]. Dimethylsulfoxide reductase (DMSOR) is the most widespread molybdoprotein family in both archaea and bacteria, which was present in more than 90% Mo-utilizing organisms (Figure 2B). MOSC-containing protein, xanthine oxidase (XO), and SO families were also widespread in the majority of Mo-utilizing bacteria; however, most sequenced archaea do not have MOSC-containing protein and XO families. Several new domain fusions were detected for different members of DMSOR, SO, and XO in prokaryotes, providing valuable information for the inference of protein interactions and functions. The Fe-Mo-containing nitrogenase was only needed by a small number of bacteria and methanogenic archaea. On the other hand, MOSC-containing protein (or named mARC), SO, and XO are the three eukaryotic molybdeoprotein families, all of which were detected in almost all organisms that use Mo, indicating that they are all critical for maintaining the function of Mo in this kingdom. With regard to molybdoproteomes, many organisms in Actinobacteria and several subclasses of Proteobacteria were molybdoprotein-rich organisms (>20 molybdoprotein genes). To date, the largest molybdoproteome in bacteria was found in *Gordonibacter pamelaeae 7-10-1-b* (73 molybdoprotein genes, mostly belonging to the DMSOR family) [83]. In contrast, very few molybdoprotein-rich organisms were observed in archaea and eukaryotes. Further examination of the relationship between environmental factors and molybdoproteins revealed that the majority of molybdoprotein families and large molybdoproteomes are more frequently present in aerobic organisms, implying that oxygen has played a crucial role in the evolution of molybdoprotein genes [83].

Although it is still very difficult to distinguish between Mo and W utilization in different members of molybdoproteins due to quite similar physical-chemical and functional properties [91], it is worth mentioning that several attempts have been made to identify tungstoproteins from molybdoprotein families based on recent advances on tungstoproteins [83,92]. The currently known tungstoproteins include nearly all enzymes of the aldehyde:ferredoxin oxidoreductase (AOR) family and certain enzymes of the DMSOR family, including formate dehydrogenase and acetylene hydratase from strictly anaerobic bacteria and formylmethanofuran dehydrogenase from methanogenic archaea [92,93,94]. Preliminary analysis of tungstoproteins in prokaryotes revealed that AOR could be detected in the majority of W-utilizing prokaryotes while W-containing DMSOR proteins were present in most W-utilizing bacteria and a small number of archaea (mainly methanogens) (Figure 2C) [83]. These exploratory studies may provide the first global view of W utilization in prokaryotes.

### 3.4. Nickel and Cobalt

Ni is an essential cofactor for several enzymes that play critical roles in energy and nitrogen metabolism [15,95]. Some other Ni-containing proteins, such as glyoxalase I and acireductone dioxygenase, are not strictly Ni-dependent proteins which may bind alternative metals in different or even same organisms [95]. Co is mainly used as a key component of cobalamin (or called vitamin B_12_), which encompasses a group of closely related corrinoid compounds found in enzymes that mediate methyl transfer reactions, isomerase rearrangements, dehalogenation, and some other processes [96,97,98]. Moreover, Co is also detected in several non-corrin Co-containing enzymes in certain organisms, which may use other metals (such as Zn and Fe) to replace Co in many other organisms [99]. In this review, we only discuss strictly Ni-dependent and B_12_-binding protein families which are shown in Table 3.

To our knowledge, only few comparative genomic studies have been conducted on Ni- or Co-dependent metalloproteins in a wide range of organisms from the three domains of life [81,100,101]. As prokaryotes use similar import systems for Ni and Co uptake [102,103], the utilization of the two trace metals could be highly correlated, which was supported by the observation that most prokaryotic organisms use both metals [81]. In bacteria, urease and methionine synthase (MetH) were the most frequently used Ni- and Co-dependent protein families, respectively (Figure 2D,E). However, they seemed to be rare or even absent in archaea, in which Ni-Fe hydrogenase and B_12_-dependent ribonucleotide reductase class II (RNR II) were the most commonly used Ni and Co enzymes. Except for a small number of organisms (such as deltaproteobacteria and Methanosarcina species), most prokaryotes possessed no more than 5 Ni- and/or Co-dependent metalloprotein genes. The largest Ni-dependent proteome was previously reported in *Deltaproteobacterium MLMS-1* (16 Ni-binding protein genes, half were Ni-Fe hydrogenases) and the largest B_12_-dependent proteome in *Dehalococcoides sp. CBDB1* (35 B_12_-dependent protein genes, 32 were reductive dehalogenase CprA proteins) [81,100]. Another recent study analyzed the distribution of vitamin B_12_ production pathway and a variety of B_12_-dependent enzymes in over 11,000 bacterial species, which provides important information on B_12_ utilization and its evolution in a much wider prokaryotic range [104]. Approximately 86% of the examined bacteria contained B_12_-dependent enzyme families, most of which lacked the ability to synthesize B_12_ and have to obtain this cofactor from exogenous sources. Proteobacteria and Bacteroidetes appeared to have larger numbers of B_12_-dependent enzymes than others.

In contrast to prokaryotes, the utilization of Ni and Co is quite restricted in eukaryotes, and very few organisms utilize both metals [81]. Only one Ni-dependent enzyme (urease) and three B_12_-dependent enzymes (methylmalonyl-CoA mutase, RNR II, and MetH) have been reported in this kingdom (Figure 2D,E). Urease and MetH were present in all Ni- and Co-utilizing eukaryotes, respectively. Analysis of Ni- and Co-dependent metalloproteomes did not reveal organisms that contained many of these proteins. Interestingly, compared to the majority of unicellular organisms that lack B_12_-binding proteins, *Dictyostelium discoideum* and several *Phytophthora* species contained all the three known eukaryotic B_12_-dependent enzymes, implying a more important role of B_12_ cofactor in these organisms [81]. In the future, it is necessary to perform more comprehensive surveys on the two metals using newly generated genomic resources.

### 3.5. Selenium

Se is a metalloid trace element, which is essential for normal physiological functions in humans, animals, and many other organisms [5,105]. It mainly occurs in the form of Sec, which is a key component of selenoproteins involved in numerous enzymatic reactions, such as redox homeostasis, thyroid hormone metabolism, anti-inflammatory actions, and reproduction [106,107]. The mechanism of Sec biosynthesis and its incorporation into proteins has been elucidated in both prokaryotes and eukaryotes [21,22]. So far, a significant number of selenoproteins have been reported in various organisms from bacteria to mammals, many of which were identified using reliable bioinformatic approaches [50,51,52,57,108,109]. Table 4 lists the majority of known and putative selenoproteins. Although the functions of most selenoproteins are not known and could only be inferred by sequence homology, it is very likely that most of them play important roles in antioxidation and detoxification [106].

**Figure 3:**
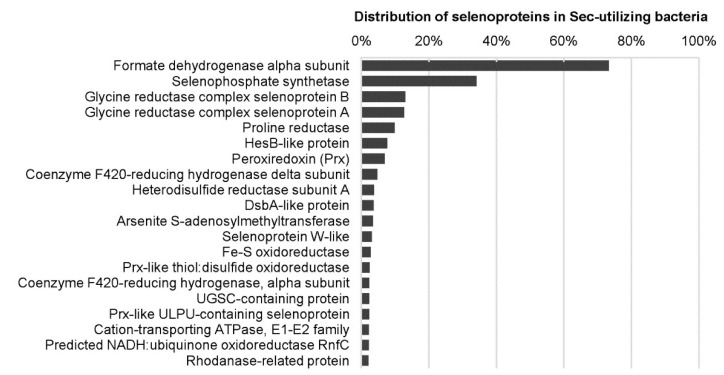
Distribution of the top 20 selenoproteins in Sec-utilizing bacteria. Data used to generate this figure can be found in [114].

Previously, several computational and comparative genomic approaches have been carried out to investigate the distribution and evolution of Se metabolic pathways and selenoproteins in a large number of prokaryotic organisms and selected environmental samples [23,81,110,111,112,113,114,115,116], which provide detailed information on how this element is selectively used by proteins and organisms from different kingdoms. An early work analyzed the Sec biosynthetic pathway and known selenoproteins in several hundred bacterial and archaeal genomes, and found that only one-fourth of the examined organisms have selenoprotein genes. Most selenoprotein-rich organisms belong to Deltaproteobacteria and Clostridia [81]. Recently, a much more extensive evaluation has been conducted on Se metabolism and selenoproteins in bacteria by analyzing more than 5200 genomes, which generated the largest map of Se utilization in this kingdom [114]. More than 60 selenoprotein families/subfamilies could be detected in bacteria. Formate dehydrogenase alpha subunit and selenophosphate synthetase were the most widespread bacterial selenoprotein families (Figure 3). A new selenoprotein-rich phylum Synergistetes and additional selenoprotein-rich organisms have also been identified. The largest bacterial selenoproteome was found in *Syntrophobacter fumaroxidans*, a syntrophic propionate-oxidizing deltaproteobacterium containing 39 selenoprotein genes [81,114]. Although both aerobic and anaerobic organisms could use Sec, the fact that most selenoprotein-rich organisms (78.3%) are obligate or facultative anaerobic suggests a somewhat stronger correlation between evolution of selenoprotein genes and low oxygen level [114].

In archaea, selenoprotein genes were only detected in a small number of organisms belonging to three phyla: Methanococcales, Methanopyrales, and Lokiarchaeota [81,116,117,118]. Compared to bacteria which contain a variety of known or predicted selenoprotein families, only nine selenoprotein families have been discovered in archaea, most of which are involved in methanogenesis [117]. The archaeal selenoproteomes show a relatively narrow size distribution (7~12 selenoproteins). Lokiarchaeota, a novel archaeal phylum and the closest archaeal relative to eukaryotes, was reported to have the largest archaeal selenoproteome (at least 12 selenoprotein genes) [118]. Further analysis of Lokiarchaeota selenoprotein genes suggests that this archaeon may serve as an intermediate form between the typical archaeal and eukaryotic Sec biosynthesis systems, providing new clues for the origin and evolution of the Sec utilization trait.

More efforts have been made to explore the distribution and evolution of selenoproteins in eukaryotes [81,119,120,121,122,123,124]. Several early comparative studies demonstrated that many selenoprotein families, such as glutathione peroxidases (GPXs), thioredoxin reductases (TXNRDs), and selenophosphate synthase 2 (SEPHS2) are shared between single-cell eukaryotes (such as green algae and many protists) and vertebrates, implying that the majority of eukaryotic selenoproteins originated from the ancestors of current eukaryotes and have been preserved throughout evolution [81,119,120,121]. However, massive and independent selenoprotein gene loss events (either loss of selenoprotein genes or replacement of Sec with Cys residue) were observed in different lineages such as fungi, land plants, nematodes, and some other organisms [119,120]. The size of eukaryotic selenoproteomes varies greatly between species. With the exception of mammals, aquatic organisms (such as algae and fish) generally have larger selenoproteomes than terrestrial ones (such as insects and nematodes). Although parallel loss of Sec utilization was observed in different groups of algae [122], the largest eukaryotic selenoproteome was described in the harmful pelagophyte alga *Aureococcus anophagefferens* (containing at least 59 selenoprotein genes) [125]. In animals, amphioxus was found to have the most abundant and diverse selenoproteins (containing 40 selenoprotein genes) [121]. Further investigation of selenoproteins in sequenced vertebrates defined the ancestral vertebrate (28 selenoproteins) and mammalian (25 selenoproteins) selenoproteomes, and reconstructed their evolutionary history [120]. For example, mammalian TXNRD1 and TXNRD3 were found to have evolved from an ancestral glutaredoxin-domain-containing enzyme, and selenoprotein V and GPX6 appeared at the root of placental mammals by duplications of selenoprotein W and GPX3, respectively. By evaluating the potential forces for selenoprotein gain or loss and for substitutions between Sec and Cys residues in different vertebrate clades, it was proposed that the strength of natural selection on selenoprotein genes is distinct between land vertebrates and teleost fishes, suggesting that Se availability has shaped the evolution of vertebrates [124]. In addition, selenoprotein P (SELENOP), the only human selenoprotein with multiple Sec residues, has been suggested to function as a genetic marker of Se utilization in animals (i.e., its number of Sec residues correlates with the selenoproteome size) [126]. A recent study showed that SELENOP genes are present across metazoan lineages with highly variable numbers of Sec-TGA codons, ranging from a single Sec residue in certain insects to up to 132 in bivalve mollusks, implying a highly dynamic evolutionary process of this selenoprotein [127]. Very recently, it was also reported that Sec could be encoded by several early-branching fungal phyla, which provides new insights into the evolution of Sec utilization in fungi [128].

Theoretically, comparative genomic approaches could be applied to study the metabolism of all trace metals and to identify the corresponding metalloproteins. However, due to limited knowledge about metal-binding sites and related properties for several other metals such as Mn, Cr, and V, metalloproteins that are strictly dependent on them remain poorly defined. For example, Mn is known to serve as a substitute for some other metals (e.g., Zn and Mg) in the active sites of numerous enzymes, resulting in the difficulty to distinguish Mn-dependent proteins from other metalloproteins [129]. Only a few proteins have been reported to bind Cr or V in certain organisms, including Cr-containing oligopeptide chromodulin and V-containing vanabins and haloperoxidases [130,131]; however, it is unclear whether these proteins are strictly dependent on the corresponding metal in other organisms. Therefore, comparative analysis of these metalloproteins seems to be a hard task and needs to be solved in the future.

## 4. Conclusions

Bioinformatics offers a powerful tool for studying metalloproteins, metalloproteomes, and their evolutionary trends in nature. Many of these studies have been devoted to the identification of metalloproteins based on known metal-binding motifs, patterns, and profiles. To date, it is still very difficult to identify complete metalloproteomes for almost all metals. Nevertheless, recent progress on bioinformatic research of metalloproteins, especially comparative genomics of several essential trace metals, has provided significant insights into the general principles of metal utilization and evolution across the three domains of life. In this review, we highlight recent studies that used various computational strategies and methods to predict metalloproteins and to investigate the distribution and evolution of metalloproteins/metalloproteomes in a wide range of organisms. In the future, with the dramatic increase in the number of sequenced genomes and improved computational techniques for identifying more metalloprotein genes, bioinformatics and comparative genomic approaches will play an even more important role in better understanding of metal utilization and function in biology.

## Figures and Tables

**Table 1:** Computational tools for metal-binding site and metalloprotein prediction.

Name	Website	Related Metals	Main Algorithm	Reported Performance	Ref.
RDGB	http://www.cerm.unifi.it/home/research/genomebrowsing.html	Zn, Cu, Fe and other metals	Integration of tools for retrieval of protein domains and genome analysis	Accuracy: 89.6%, precision: 85.9%	[32]
Zincfinder	http://zincfinder.dsi.unifi.it	Zn	^a^ SVM	^b^ AURPC: 0.590 (local predictor) and 0.633 (gating network)	[33]
Zincpred	http://www.fos.su.se/~nanjiang/zincpred/download/	Zn	SVM- and homology-based algorithm	AURPC: 0.723 (local predictor) and 0.701 (gating network)	[34]
TEMSP	http://netalign.ustc.edu.cn/temsp/	Zn	Structure-based algorithm with a range of geometric criteria	^c^ AUC: 0.945	[35]
Zincidentifier	http://protein.cau.edu.cn/zincidentifier/	Zn	A two-step feature selection method based on random forest algorithm	AUC: 0.955, AURPC: 0.829	[36]
ZincExplorer	http://protein.cau.edu.cn/ZincExplorer/	Zn	A combination of SVM-, cluster- and template-based predictors	AURPC: 0.907	[37]
ZincBinder	http://proteininformatics.org/mkumar/znbinder/	Zn	SVM model trained on PSSM-based input feature	AUC: 0.91	[38]
ZINCCLUSTER	http://www.metalactive.in	Zn	SVM-based Ligand Finder and Cluster Finder algorithms	^d^ MCC: 0.798, F1-score: 0.801	[39]
ZnMachine	http://bioinformatics.fzu.edu.cn/znMachine.html	Zn	A combination of several intensively-trained machine learning models	AUC: 0.933 (SVM) and 0.910 (neural network)	[40]
HemeBIND	http://mleg.cse.sc.edu/hemeBIND/	Fe (heme)	SVM	MCC: 0.504, F1-score: 56.87%	[41]
SCMHBP	http://iclab.life.nctu.edu.tw/SCMHBP/	Fe (heme)	Based on a newly-developed scoring card method for predicting heme-binding proteins	Accuracy: 85.90%	[42]
Isph	http://biodev.extra.cea.fr/isph	Fe (Fe-S)	A penalized linear model based on machine learning approach	Precision: 87.9%, recall: 80.1% (extended model)	[43]
MetalPredator	http://metalweb.cerm.unifi.it/tools/metalpredator/	Fe (Fe-S)	Integration of existing domain-based methodology with a new approach for discovering metal-binding motifs	Precision: 85.2%, recall: 88.6%	[44]
MetSite	N/A	Fe, Zn, Cu, Mn, Ca, Mg	Artificial neural network	Mean accuracy: 94.5%	[45]
FINDSITE-metal	http://cssb.biology.gatech.edu/findsite-metal/	Fe, Zn, Cu, Mn, Ni, Co, Ca, Mg	Integration of structure/evolutionary information and machine learning approach (SVM)	Overall accuracy: 70–90%	[46]
SeqCHED	http://ligin.weizmann.ac.il/seqched	Fe, Zn, Cu, Mn, Ni, Co, Ca, Mg	A modification of the CHED algorithm and machine learning filters (decision tree classifier and SVM)	Sensitivity: 84–85%, selectivity: 82–93% (stringent filtration)	[47]
MetalDetector	http://metaldetector.dsi.unifi.it/v2.0/	Transition metals that use cysteine and histidine as ligands	A combination of different machine learning algorithms (SVM-HMM, structured-output SVM)	Precision: 60–79%, recall: 71–88%	[48]
MIB	http://bioinfo.cmu.edu.tw/MIB/	Ca, Cu, Fe, Mg, Mn, Zn, Cd, Ni, Hg, Co	Fragment transformation method	Overall accuracy: 92.9–95.1%	[49]
SECISearch3 and Seblastian	http://seblastian.crg.eu/, http://gladyshevlab.org/SelenoproteinPredictionServer	Se	Homology-based RNA motif finding and selenoprotein gene detection approach	Precision: 81.48–100%, recall: 33.33–100%	[50]
SelGenAmic	N/A	Se	Selenoprotein gene assembly algorithm based on the GenAmic approach used by geneid	N/A	[51]
bSECISearch	http://genomics.unl.edu/bSECISearch/	Se	An algorithm for prediction of bacterial selenoprotein genes based on a concensus RNA structural model	True positive rate: 96.5%	[52]

^a^ SVM: support vector machine. ^b^ AURPC: area under the recall-precision curve. ^c^ AUC: area under the curve. ^d^ MCC: Matthew’s correlation coefficient.

**Table 2:** Metalloprotein databases.

Name	Website	Main Content	Ref.
MDB	http://metallo.scripps.edu	Metalloproteins and metal-binding sites in protein structures	[58]
Metal-MACiE	http://www.ebi.ac.uk/thornton-srv/databases/Metal_MACiE/home.html	All metalloenzymes annotated in the MACiE database	[59]
dbTEU	http://gladyshevlab.bwh.harvard.edu/trace_element/	Transporters and metalloproteins for Cu, Mo, Co, Ni, and Se in more than 700 organisms	[60]
Mespeus	http://mespeus.bch.ed.ac.uk/MESPEUS_10/	Experimentally established geometry of metal and protein interactions	[61]
MetalPDB	http://metalweb.cerm.unifi.it	Metal-binding sites detected in the 3D structures of biological macromolecules	[62]
SelenoDB	http://www.selenodb.org	Selenoprotein genes in at least 58 animal genomes	[63]
ZincBind	http://zincbind.bioinf.org.uk	All known Zn-binding sites from PDB	[64]

**Table 3:** List of known metalloprotein families for several metals in prokaryotes and eukaryotes.

Metal	Prokaryotes	Eukaryotes
Cu	Cytochrome c oxidase subunit I	Cytochrome c oxidase subunit I
	Cytochrome c oxidase subunit II	Cytochrome c oxidase subunit II
	Plastocyanin family	Plastocyanin family
	Cu amine oxidase	Cu amine oxidase
	Cu-Zn superoxide dismutase	Cu-Zn superoxide dismutase
	Multicopper oxidase family	Multicopper oxidase family
	Tyrosinase	Tyrosinase
	Azurin	Galactose oxidase
	Rusticyanin	Hemocyanin
	Nitrosocyanin	Plantacyanin family
	Nitrous oxide reductase	Peptidylglycine α-hydroxylating monooxygenase
	Nitrite reductase	Dopamine β-monooxygenase
	NADH dehydrogenase 2	Cnx1G
	Particulate methane monooxygenase	
Mo	Sulfite oxidase	Sulfite oxidase
	Xanthine oxidase	Xanthine oxidase
	Dimethylsulfoxide reductase	MOSC-containing protein (mARC)
	MOSC-containing protein	
	Fe-Mo-containing nitrogenase	
W	Aldehyde:ferredoxin oxidoreductase	N/A
	Certain members of dimethylsulfoxide reductase:	
	Formate dehydrogenase and acetylene hydratase (obligately anaerobic bacteria)	
	Formylmethanofuran dehydrogenase (methanogenic archaea)	
Ni	Urease	Urease
	Ni-Fe hydrogenase	
	Carbon monoxide dehydrogenase	
	Superoxide dismutase SodN	
	Acetyl-coenzyme A synthase/decarbonylase	
	Methyl-coenzyme M reductase	
	Lactate racemase	
Co	Methylmalonyl-CoA mutase	Methylmalonyl-CoA mutase
	Isobutyryl-CoA mutase	B_12_-dependent ribonucleotide reductase class II
	Ethylmalonyl-CoA mutase	Methionine synthase
	Glutamate mutase	
	Methyleneglutarate mutase	
	D-lysine 5,6-aminomutase	
	Diol dehydratase	
	Glycerol dehydratase	
	Ethanolamine ammonia lyase	
	B_12_-dependent ribonucleotide reductase class II	
	Methionine synthase	
	Methyltetrahydromethanopterin:coenzyme M methyltransferase subunit A	
	Other methyltransferases	
	B_12_-dependent reductive dehalogenase PceA/CprA	
	LitR/CarH/CarA	
	PpaA	
	Epoxyqueuosine reductase	

**Table 4:** List of known selenoprotein families.

Prokaryotes	Eukaryotes
***Known selenoproteins***	***Known selenoproteins in mammals***
Formate dehydrogenase alpha subunit	Deiodinase (DIO) family: DIO1, DIO2, and DIO3
Selenophosphate synthetase	Glutathione peroxidase (GPX) family: GPX1, GPX2, GPX3, GPX4, and GPX6
Coenzyme F420-reducing hydrogenase alpha subunit	Thioredoxin reductase (TXNRD) family: TXNRD1, TXNRD2, and TXNRD3
Coenzyme F420-reducing hydrogenase delta subunit	Methionine sulfoxide reductase B1
Methylviologen-reducing hydrogenase alpha subunit	Selenoprotein F
Glycine reductase selenoprotein A	Selenoprotein H
Glycine reductase selenoprotein B	Selenoprotein I
Proline reductase	Selenoprotein K
Heterodisulfide reductase alpha subunit	Selenoprotein M
Methionine-S-sulfoxide reductase	Selenoprotein N
Peroxiredoxin (Prx)Thioredoxin (Trx)	Selenoprotein O
Glutaredoxin (Grx)	Selenoprotein P
Arsenite S-adenosylmethyltransferase	Selenoprotein S
	Selenoprotein T
***Predicted selenoproteins:***	Selenoprotein V
Thiol:disulfide isomerase-like protein	Selenoprotein W
Thiol:disulfide interchange protein	Selenophosphate synthetase 2
HesB-like	
Deiodinase-like	***Other known selenoproteins:***
Glutathione peroxidase-like	Methionine-S-sulfoxide reductase
Selenoprotein W-like	Protein disulfide isomerase
Fe-S oxidoreductase	Selenoprotein J
DsbA-like	Selenoprotein L
DsrE-like	Selenoprotein U
DsbG-like	Selenoprotein E
AhpD-like	SAM-dependent methyltransferase
Arsenate reductase	
Molybdopterin biosynthesis protein MoeB	***Predicted selenoproteins:***
Glutathione S-transferase	Prx-like protein
COG0737 UshA	Trx-fold protein
OsmC-like	Membrane selenoprotein MSP
Rhodanase-related protein	SelTryp
Sulfurtransferase COG2897	Other hypothetical proteins
Cation-transporting ATPase, E1-E2 family	
Methylated-DNA-protein-cysteine methyltransferase	
UGSC-containing protein	
CMD domain containing protein	
Organic mercuric lyase MerB2	
Predicted redox-active disulfide protein 2	
Prx-like/Trx-like/Grx-like and Trx-fold proteins	
Other hypothetical selenoproteins	
